# Endoscopic Ultrasound Detection of Type B Aortic Dissection

**DOI:** 10.14309/crj.0000000000000639

**Published:** 2021-07-21

**Authors:** Sindhura Kolli, Kruthika Bachali, Daryl Ramai, Vahe Shahnazarian, Denzil Etienne, Madhavi Reddy, Krishna C. Gurram

**Affiliations:** 1 Division of Internal Medicine, NYU Grossman School of Medicine, New York, NY; 2 Division of Gastroenterology and Hepatology, The Brooklyn Hospital Center, Brooklyn, New York, NY; 3 Department of Gastroenterology and Hepatology, Elmhurst Hospital, Queens, New York, NY

## CASE REPORT

**Figure 1.**
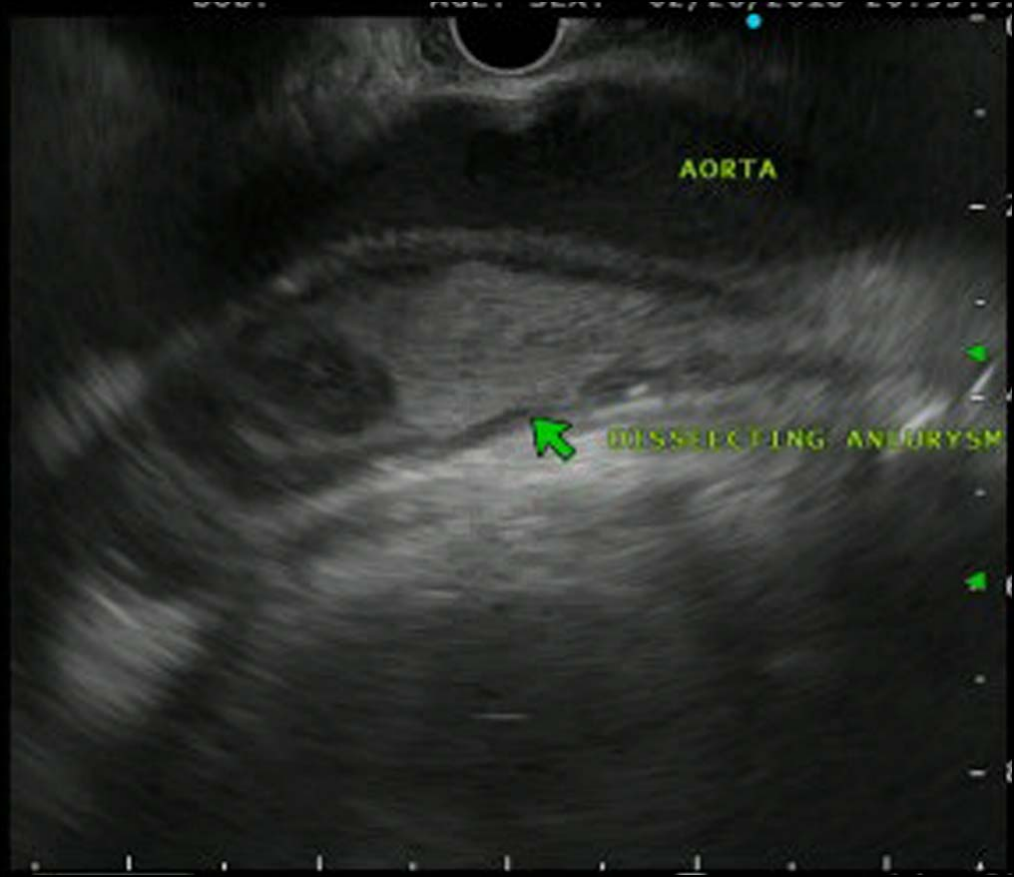
Type B aortic dissection as seen on endoscopic ultrasound.

A 74-year-old woman with no medical history presented to the emergency department with postprandial unrelenting chest pain radiating from the right breast to the right upper abdominal quadrant and accompanied by nausea. Physical examination was significant for a tender mass palpated in the right upper and lower quadrants. Right upper quadrant ultrasound displayed choledocholithiasis with common bile duct dilation at 1.1 cm, unchanged from 1 year earlier, and sludge in the gallbladder without cholecystitis. Vitals were within normal limits with the exception of raised blood pressure of 149/67 mm Hg, likely secondary to pain. Endoscopic ultrasonography (EUS) examination confirmed the choledocholithiasis, sludge, common bile duct dilation, and thickened gallbladder wall. Incidentally, it also revealed a type B dissection of the thoracic aorta and aortic arch (Figure 1).

An aortic dissection (AD) occurs secondary to injury to the intima and media layers of the aorta. Location dictates categorization, with type A AD occurring in the ascending aorta, while type B AD occurring in the descending portion. Traditionally, computed tomographies with contrast dye, transesophageal echocardiogram, and/or magnetic resonance images are commonly used to diagnose AD.^1,2^ However, when patients present asymptomatically or atypically or present with a predominant gastrointestinal complaint, a comprehensive EUS examination that includes cardiac structures could provide an alternative method of early detection and treatment of cardiac diseases. The endoscope is introduced into the esophagus similarly to a transthoracic echocardiogram and scans from 120° to 180° and can be flexed anteriorly for detailed imaging. Its limitations are a poor visualization of right-sided cardiac structures, no retroflexion or lateral flexion options, detection of low flow rates rather than the high-velocity cardiac flow rates, and have less than half the frame rate of a transesophageal echocardiogram. However, it allows for a thorough and precise visualization of the aortic valve, the mitral valve, ascending and descending aorta, pericardium, left atrial appendage, and interatrial septum because of these structures' proximity to the esophagus.^3^ In this manner, we hope to widen the utility of an EUS to capture critical findings that would be otherwise missed.

## DISCLOSURES

Author contributions: All authors contributed equally to this manuscript. KC Gurram is the article guarantor.

Financial disclosure: None to report.

Previous presentation: This case was presented at the American College of Gastroenterology Annual Scientific Meeting; October 5-10, 2018; Philadelphia, Pennsylvania.

Informed consent was obtained for this case report.
